# Mortality prognostic factors in acute pancreatitis

**Published:** 2016

**Authors:** CC Popa, DC Badiu, OC Rusu, VT Grigorean, SI Neagu, CR Strugaru

**Affiliations:** * “Carol Davila” University of Medicine and Pharmacy, Bucharest, Romania; ** Department of Surgery, 2nd General Surgery Clinic, University Emergency Hospital, Bucharest, Romania; *** Department of Surgery, General Surgery Clinic, “Bagdasar-Arseni” Clinical Emergency Hospital, Bucharest, Romania; **** Department of Medical Genetics, “Carol Davila” University of Medicine and Pharmacy, Bucharest, Romania

**Keywords:** acute pancreatitis, mortality, markers, prognostic

## Abstract

**Background:** The aim of the study was to present the biological prognostic factors of mortality in patients with acute pancreatitis.

**Methods:** Several usual laboratory values were monitored: glucose, urea, partial pressure of oxygen, WBC count, hemoglobin, total bilirubin, and cholesterol. A statistical analysis was performed by using ROC curves and AUC interpretation.

**Results:** The overall mortality rate was 21.1% and was different depending on the severity of the disease. Only 2.22% of the patients with a mild disease died, as opposed to 45.63% of the patients with a severe form. All the analyses studied were significantly elevated in the deceased patients. A close correlation between blood glucose, urea, partial pressure of oxygen, WBC, hemoglobin, total bilirubin, and cholesterol and mortality was objectified by measuring the AUC, which was of 97.1%, 95.5%, 93.4%, 92.7%, 87.4%, 82.2%, and 79.0%.

**Conclusions:** The usual, easy to use, fast, and cheap tests were useful in predicting mortality in patients with acute pancreatitis. Our study confirmed that the combination of several factors led to an accurate mortality prediction.

## Introduction

The majority of patients diagnosed with acute pancreatitis developed mild or moderate forms. A smaller group of patients developed severe forms, characterized by the development of multiple system organ failure and/or necrotic changes of the pancreas and peripancreatic forms that were accompanied by increased morbidity and mortality.

It is therefore important to assess the severity of acute pancreatitis as early as possible. Studies in recent years have highlighted numerous prognostic factors for mortality in acute pancreatitis.

The aim of this study was to highlight possible prognostic biological factors of mortality in acute pancreatitis.

## Material and methods

The study was prospective and was conducted over a period of four years between 2007 and 2010 in the General Surgery Clinic of “Bagdasar-Arseni”Clinical Emergency Hospital, Bucharest.

The data of 238 patients admitted and diagnosed with acute pancreatitis or its complications were collected from the observation charts of patients.

The diagnosis of acute pancreatitis was established based on clinical results, laboratory tests, and ultrasounds and/or abdominal computed tomography.

The assessment of severity of acute pancreatitis was performed based on clinical, biological, imaging, pathological, and intraoperative criteria.

Values of blood biological constants were monitoredin the conductedstudy: WBC count, hemoglobin, glycemia, urea, total bilirubin, pO2, and total cholesterol.

Statistical analysis was performed by including monitored variables to observe the impact of the biological risk factors on mortality in acute pancreatitis. SPSS statistical software for Windows 10.0 was used to coordinate values and statistical tests. A comparison of the results between the group of patients who survived and the group of patients who died was performed by using t-test, contingency tables, and Pearson’s chi-square test. Spearman’s correlation coefficient was used for the possible correlations. Data were expressed as numbers, averages, standard deviations, ranges, and percentages. The value of p <0.05 was considered statistically significant. ROC curve (Receiver Operation Characteristics) was performed to determine the accuracy of diagnosis of laboratory tests by using Area Under Curve (AUC) for the respective analysis.

## Results

Out of the 238 patients in the study group, most patients presented a mild form of the disease (135 patients representing 56.72%) and only 103 patients (43.28%) developed a severe form of acute pancreatitis.

**Fig. 1:**
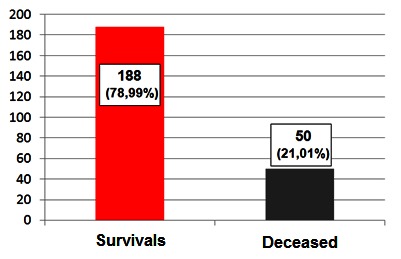
Distribution of patients according to the survival rate

**Fig. 2:**
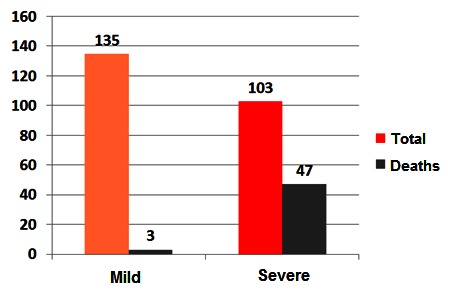
Distribution of patients according to the severity and mortality rates

The number of patients who survived was 188 (78.99%) and 50 patients died (21.01%) (**Fig. 1**). Mortality was different, depending on the severity of the disease. In the case of 135 patients who developed a mild form of the disease, only 3 patients died (2.22%), in contrast to the 47 patients who died among those 103 patients with a severe form (45.63%) (**Fig. 2**).

The performance achieved by biochemical tests was evaluated in the classification of the two classes of patients, by using ROC curves: survivors or deceased.

**Fig. 3:**
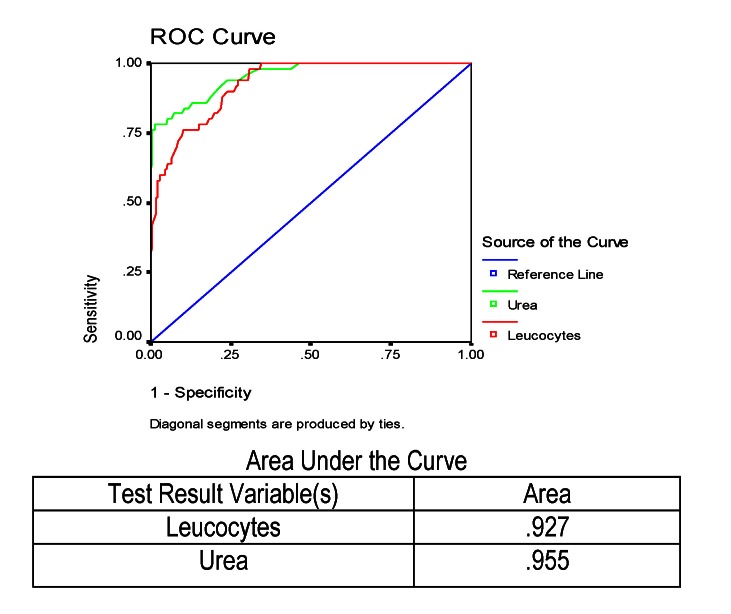
ROC and AUC results for leukocytes and urea in predicting mortality in severe acute pancreatitis

The results of urea and WBC were studied. These analyses were measured in all the patients who died, reported to the total number of patients investigated, the ROC curve for the two analyses being presented graphically in **Fig. 3**.

It was noted that these two variables were comparable regarding the small specificities. As opposed, in the case of high specificities, but also on the whole, the amount of urea was better (area under the curve was 95.5% versus only 92.7% in the number of leukocytes). After that result, we could say that urea better identified the patient who would die.

**Fig. 4:**
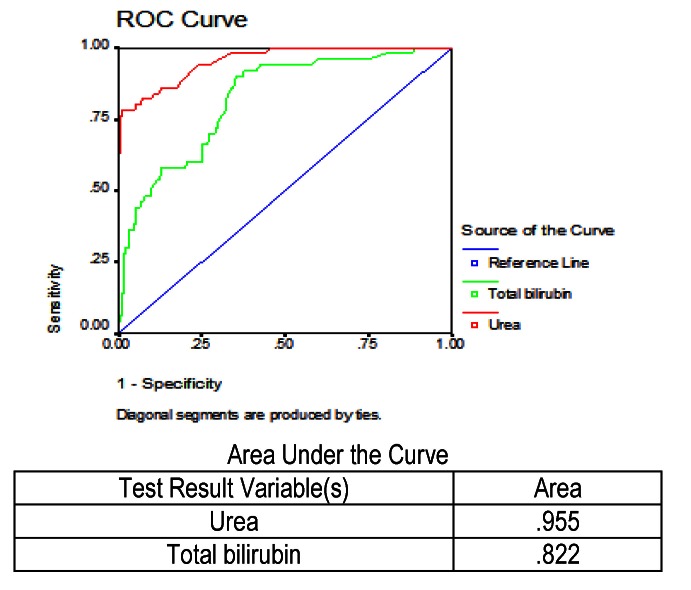
ROC and AUC results for urea and total bilirubin in predicting mortality in severe acute pancreatitis

In the next stage, we studied urea and total bilirubin values, which were measured in all patients who died or who survived. The ROC curve for urea and total bilirubin is shown in the figure below (**Fig. 4**). Analyzing the figure, we observed that between the two parameters studied, urea, which had an area under the curve of 95.5%, compared to total bilirubin, which had only 82.2%, would better identify patients who would die.

**Fig. 5:**
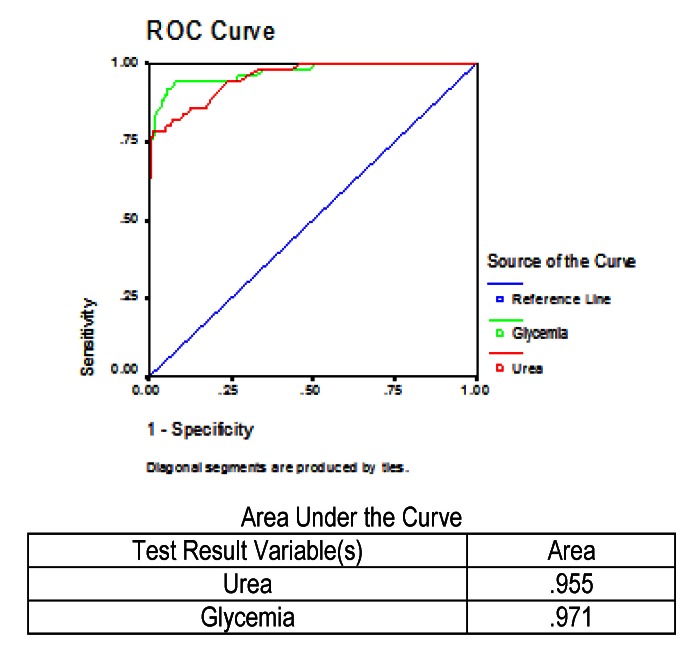
ROC and AUC results for urea and glycemia in predicting mortality in severe acute pancreatitis

Values of two other biochemical analyses, glycemia, and urea respectively, were analyzed. The ROC curve was performed for these two tests, being measured in all patients who died and reported in all patients, and the result can be observed in **Fig. 5**. 

It was noted that in the case of low specificities and also in the case of high specificities, values of the two tests were similar. What was also observed was that the value of glycemiawas better at the intermediate specificities. The area under the curve for glycemiawas 97.1%, and in the case of urea was 95.5%, thus glycemia levels were useful in predicting which patient would die.

**Fig. 6:**
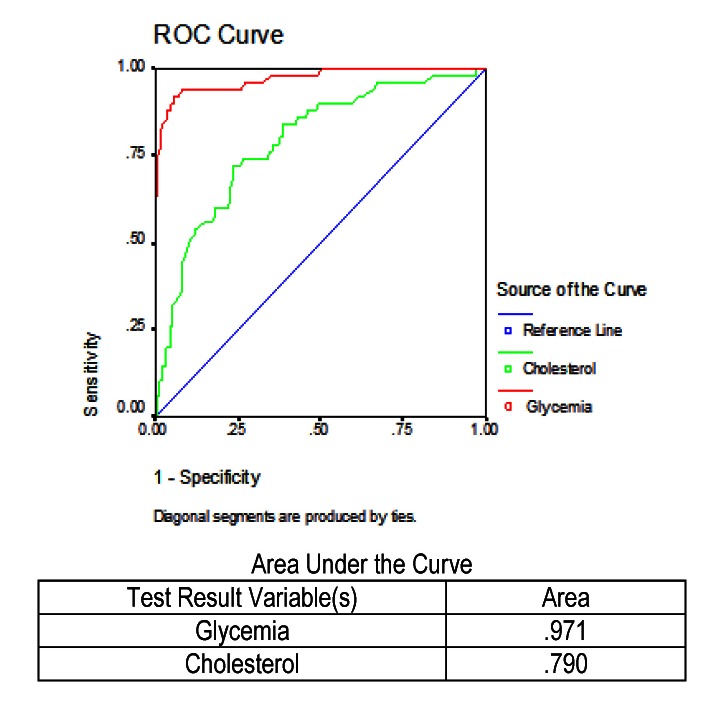
ROC and AUC results for glycemia and cholesterol in predicting mortality in severe acute pancreatitis

Another comparison was made between cholesterol and glycemia levels, an analysis measured in all the patients who died and the report was applied to the whole study group. The results obtained are shown in **Fig. 6**.

The area under the curve ofglycemia values was 97.1%, much higher than the area under the curve of cholesterol, which was only 79.0%, with the result that glycemia would identify best the mortality rates of patients.

**Fig. 7:**
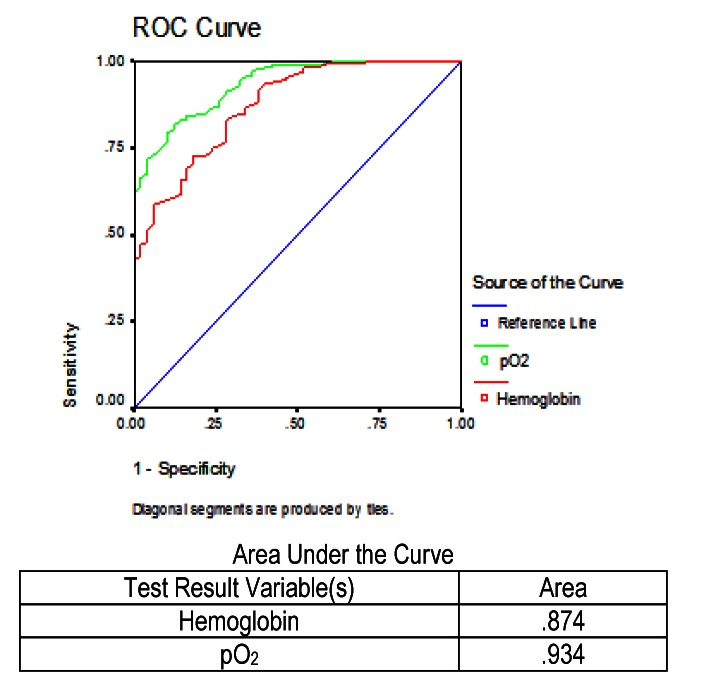
ROC and AUC results for hemoglobin and partial pressure of oxygen in the prediction of mortality from severe acute pancreatitis in patients who died

The next picture shows the ROC curve for hemoglobin and partial pressure of oxygen, measured in all patients who died (**Fig. 7**). The area under the curve in the case of hemoglobin was 87.4%, and the area under the curve for the oxygen partial pressure was 93.4%. Of the two parameters studied, we observed that the partial pressure of oxygen would better identify patients who would die.

**Fig. 8:**
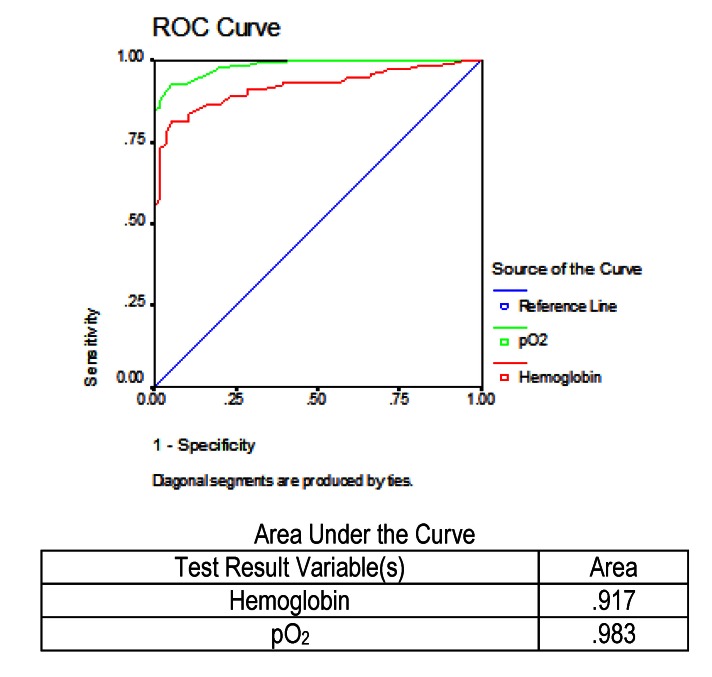
ROC and AUC results for hemoglobin and pO2 in predicting mortality in severe acute pancreatitis in patients who survived

On the other hand, the values prior to the testing of the two parameters were the same, respectively hemoglobin and the partial pressure of oxygen, but in the case of the surviving patients, it was necessary to identify patients with an increased risk of death. In the study group, 188 patients were survivors, of whom 56 patients had a severe disease. The identification of those who might die was made in the figure below, by performing the ROC curve (**Fig. 8**). It was observed that for the prediction of mortality, the values of partial pressure of oxygen were more reliable than hemoglobin.

## Discussions

In our study, mortality among patients with acute pancreatitis was relatively high (21%), similar to the results of studies from other Eastern European countries [**1**].

The type of disease severity has had a major influence on mortality. Patients with severe acute pancreatitis had a more increased mortality rate than patients with mild acute pancreatitis (45.63% vs. 2.22%). Moreover, the recent Japanese study showed similar values in terms of increased mortality in patients with severe acute pancreatitis [**2**].

In acute pancreatitis, the production mechanism of death is different along the course of the disease. Early death (in the first two weeks of hospitalization) is due to MSOF development. As opposed, late death is due to local complications assigned by pancreatic necrosis together with MSOF [**3**-**7**].

To achieve our study we performed some usual, easy harvested, reliable, and inexpensive laboratory analyses, whose results appeared quickly and could be used easily in any clinic. In all studied blood tests, we found significantly elevated changes in patients who died compared to the results obtained in the surviving patients.

The elevated values of glycemia correlated significantly with an increased mortality rate in patients with acute pancreatitis, a fact confirmed by international studies [**7**-**9**]. Several prognostic scores of acute pancreatitis also included glycemia (Ranson) in their composition, among multiple other parameters [**10**]. Representing a very important element in the prognosis of acute pancreatitis, Hong Kong score is based only on glycemia and urea, but assesses the prognosis of the disease very accurately, like in other scores, in which the assessment is more complicated [**11**]. The significant increase of glycemia in patients who died was due to endocrine pancreas damage, consequence of pancreatic structural changes in the course of the disease.

Increased urea was associated with an increased mortality, as confirmed by other studies [**7**,**12**,**13**]. Recent data showed that urea is the most important marker for predicting mortality [**14**]. On admission, increased urea is due to intravascular volume decrease, the loss of fluid and the development of prerenal azotemia [**7**,**15**]. Releasing vasoactive compounds, enzymes, and cytokines from the necrotic pancreatic tissue in the systemic circulation can cause acute kidney failure. In addition, decreased renal blood flow, hypovolemia, disseminated intravascular coagulation activation and infection increases the development of acute renal failure in these patients [**7**,**16**]. If the course of the disease progresses, it can establish multiple organ and system failure, the main cause of death [**3**,**6**]. Severity scores of acute pancreatitis in their composition are based on high levels of urea (Ranson, Glasgow/ Imrie, POP, BISAP) [**10**,**17**-**19**]. Hong Kong score is based only on two constants, urea, and glycemia [**11**].

Another parameter whose low values correlated with increased mortality is the partial pressure of oxygen. A decrease in the level of pO2 represents an important prognostic factor for mortality in relation to the entire group of studied patients. Prognostic scores have in their composition the partial pressure of oxygen (Ranson, Glasgow/ Imrie, APACHE II, SOFA, POP, JSS) [**10**,**17**,**18**,**20**-**22**]. This may be due to the imposition of acute respiratory failure, component of MSOF.

The increases in leukocyte count correlated with increased mortality, is a fact noted also by other authors [**23**]. Moreover, many prognostic scores analyses increased leukocyte count (Ranson, Glasgow, BISAP) [**10**,**17**,**19**]. The emergence and extent of pancreatic necrosis will lead to the development of early SIRS, explaining the increased leukocyte count in patients who died [**7**].

Hemoglobin, another lab test used, may reflect changes in intravascular volume status, an aspect taken into account in case of a cardiovascular dysfunction. In our study, we found a decrease in hemoglobin correlated with increased mortality. International studies have shown only a low correlation between hemoglobin (considered a surrogate for hematocrit) and mortality [**15**].

Increased total bilirubin was taken into consideration when calculating scores prognosis of acute pancreatitis, to monitor possible liver failure (SOFA) [**21**,**24**,**25**]. An increase in total bilirubin and liver disease marker within the multiple organ dysfunction syndrome was observed in the deceased patients in our study. The main cause of acute pancreatitis was a gallstone in all its forms, anatomic and clinical. The clinical implication of cholesterolosis was not precisely determined, but there are studies that deal with the role it plays in the pathophysiology of pancreatitis as well as the relationship with the presence of hypercholesterolemia [**26**]. The analysis of our study identified a correlation between increased cholesterol and increased mortality, possibly due to several mechanisms: direct hepatic impairment by modifying the production of bile, dyslipidemia with vascular impairment, the existence of recurrent episodes of acute pancreatitis.

## Conclusion

 There are many clinical laboratory prognostic scores in the evolution of acute pancreatitis in the specialty literature, each of these scores having its advantages and disadvantages. Our study confirmed that there is not one sure prognostic factor for mortality in patients with acute pancreatitis and only an association between several factors can lead to an accurate mortality prediction.

Although numerous modern factors have been identified for the prediction of mortality (copeptin, TRX-1, Ang-2, E-2) in patients with acute pancreatitis, such as glycemia and urea, the partial pressure of oxygen and white blood cell count tests remain easy to use, inexpensive, and accurate.

More studies should be undertaken, respectively the inclusion of other future parameters is needed to help us understand clearly the pathogenesis of acute pancreatitis and to establish the appropriate therapy as soon as possible, to improve the evolution of patients with acute pancreatitis.
